# Rescue transseptal percutaneous mitral balloon valvuloplasty for early bioprosthetic mitral valve thrombosis during VA-ECMO support: a case report

**DOI:** 10.1093/ehjcr/ytag628

**Published:** 2026-08-28

**Authors:** Aida Feu Masdemont, Júlia Pascual Mayans, Teresa de la Torre Úbeda, Andrea Virginia Monastyrski, Eduardo Andrés Astrosa Martín

**Affiliations:** Department of Cardiology, Hospital Universitari de Girona Doctor Josep Trueta, Avinguda de França, S/N, Girona 17007, Spain; Department of Cardiology, Hospital Universitari de Girona Doctor Josep Trueta, Avinguda de França, S/N, Girona 17007, Spain; Department of Cardiology, Hospital Universitari de Girona Doctor Josep Trueta, Avinguda de França, S/N, Girona 17007, Spain; Department of Cardiology, Hospital Universitari de Girona Doctor Josep Trueta, Avinguda de França, S/N, Girona 17007, Spain; Department of Cardiology, Hospital Universitari de Girona Doctor Josep Trueta, Avinguda de França, S/N, Girona 17007, Spain

**Keywords:** Bioprosthetic valve thrombosis, Mitral valve thrombosis, Mitral balloon valvuloplasty, VA-ECMO, Case report

## Abstract

**Background:**

Early bioprosthetic mitral valve thrombosis (BPVT) is a rare but life-threatening cause of acute prosthetic obstruction after mitral valve surgery. Management is particularly challenging in postcardiotomy shock requiring veno-arterial extracorporeal membrane oxygenation (VA-ECMO), where low native cardiac output and left-sided stasis may promote thrombosis despite systemic anticoagulation.

**Case summary:**

A 74-year-old man underwent bioprosthetic mitral valve replacement, tricuspid annuloplasty, pulmonary vein isolation, and left atrial appendage closure for severe primary mitral regurgitation, with intra-operative identification of ascending aortic dissection requiring replacement of the ascending aorta and hemiarch. Because of severe postcardiotomy shock with biventricular dysfunction, peripheral VA-ECMO was initiated. On post-operative Day 2, transoesophageal echocardiography demonstrated severe bioprosthetic mitral obstruction with restricted leaflet motion and a mean transmitral gradient of 25 mmHg. Repeat imaging showed persistent obstruction with extensive left atrial thrombus despite anticoagulation optimization. Surgical reintervention and systemic fibrinolysis were considered prohibitive. After Heart Team discussion, rescue transseptal percutaneous mitral balloon valvuloplasty was performed under cerebral embolic protection using a 26-mm Inoue balloon. Immediate post-procedural imaging showed improved leaflet mobility and reduction of the mean transmitral gradient from 25 to 4 mmHg, without significant mitral regurgitation. The patient was successfully weaned from VA-ECMO and remained clinically stable at 2-month follow-up.

**Discussion:**

This case illustrates that, in carefully selected critically ill patients with catastrophic early BPVT during VA-ECMO support, rescue transseptal balloon dilatation may be considered as an exceptional salvage strategy when surgery and fibrinolysis are not viable. In highly selected patients with early BPVT causing severe haemodynamic compromise during VA-ECMO support, transseptal balloon dilatation may serve as a bridge-to-recovery option when surgery and fibrinolysis are contraindicated or carry prohibitive risk.

Learning points

- Early BPVT should be considered in patients with abrupt prosthetic mitral obstruction during postcardiotomy VA-ECMO support.
- In catastrophic early BPVT, both reoperation and fibrinolysis may be contraindicated because of recent surgery, haemodynamic instability, and major embolic or bleeding risk.
- Rescue transseptal balloon dilatation may be considered as an exceptional salvage strategy in carefully selected critically ill patients when conventional options are not feasible.

## Introduction

Early bioprosthetic mitral valve thrombosis (BPVT) is an uncommon but potentially life-threatening cause of acute prosthetic obstruction after mitral valve replacement.^1^ The risk may be amplified in postcardiotomy shock requiring veno-arterial extracorporeal membrane oxygenation (VA-ECMO), where low native cardiac output and left-sided stasis promote thrombus formation despite systemic anticoagulation.^1–3^ Management is especially challenging when surgery and fibrinolysis are contraindicated. Current ESC/EACTS guidelines support anticoagulation as first-line treatment for stable bioprosthetic valve thrombosis, while surgery or fibrinolysis may be considered in unstable patients, depending on clinical context.^4^ We describe the use of rescue transseptal percutaneous mitral balloon valvuloplasty (PMBV) with cerebral embolic protection in a patient with catastrophic early BPVT during VA-ECMO support.

## Summary figure

**Figure:**
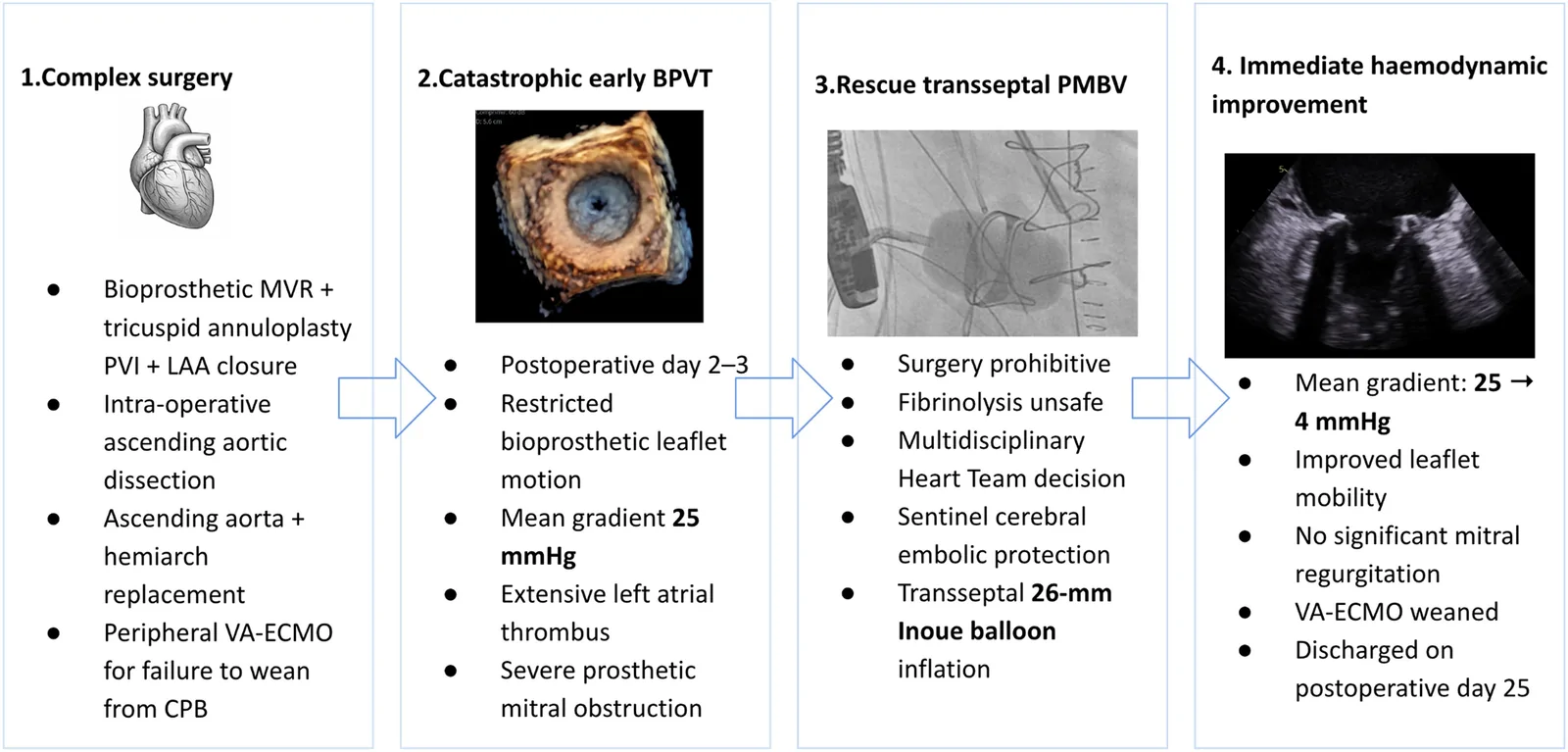


## Case presentation

A 74-year-old man with hypertrophic cardiomyopathy, reduced left ventricular ejection fraction (35%–40%), severe primary mitral regurgitation due to chordal rupture, and moderate functional tricuspid regurgitation with annular dilatation was referred for surgery after admission with acute heart failure and new-onset atrial fibrillation.

He underwent mitral valve replacement with a 29-mm Carpentier Magna Ease bioprosthesis, tricuspid annuloplasty, pulmonary vein isolation, and left atrial appendage closure. Intra-operatively, an ascending aortic dissection was identified, requiring replacement of the ascending aorta and hemiarch. Because of severe postcardiotomy shock with biventricular dysfunction and failure to wean from cardiopulmonary bypass, peripheral VA-ECMO was initiated.

**Figure 1:**
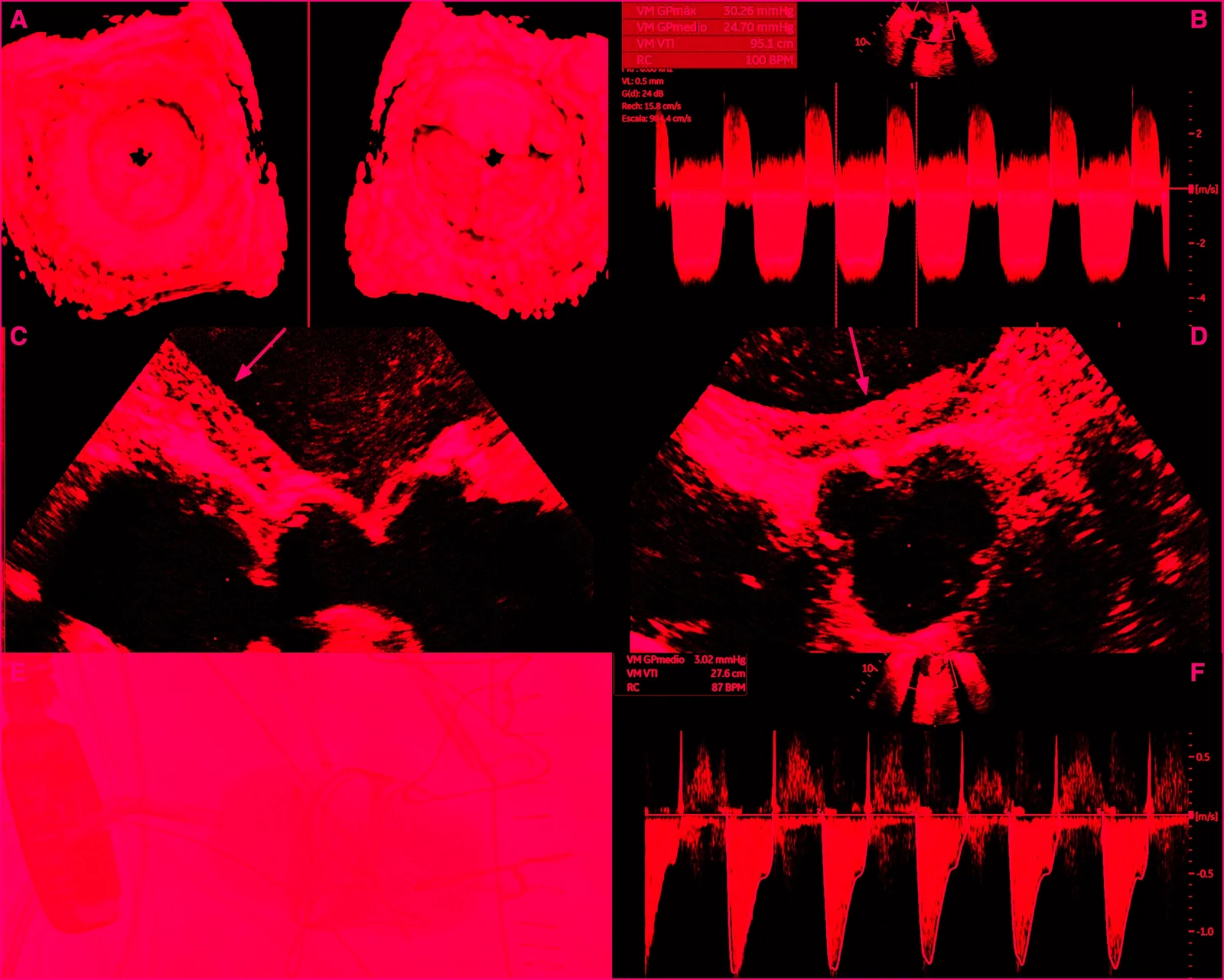
Multimodality imaging of acute bioprosthetic mitral valve thrombosis and rescue transseptal percutaneous mitral balloon valvuloplasty. (*A*) Two complementary three-dimensional transoesophageal echocardiographic views demonstrating restricted opening of the mitral bioprosthesis. (*B*) Pre-procedural continuous-wave Doppler showing severe transprosthetic mitral obstruction with a mean transmitral gradient of 25 mmHg. (*C* and *D*) Transoesophageal echocardiography demonstrating extensive mural thrombotic material within the left atrium, extending to the prosthetic annulus and surgically excluded left atrial appendage (arrows), measuring up to 10 mm in thickness. (*E*) Fluoroscopic image showing Inoue balloon inflation across the mitral bioprosthesis during transseptal balloon dilatation. (*F*) Post-procedural continuous-wave Doppler demonstrating marked reduction in transprosthetic gradient following balloon dilatation.

On admission to the cardiac intensive care unit, he remained critically ill with haemodynamic instability and multiorgan dysfunction. Unfractionated heparin was started according to the institutional VA-ECMO protocol, targeting an activated partial thromboplastin time of 60–80 s and an anti-factor Xa activity of 0.3–0.5 IU/mL, which were achieved within 24 h. On post-operative Day 2, transoesophageal echocardiography (TOE) showed severe bioprosthetic mitral obstruction with restricted leaflet motion and a mean transmitral gradient of 25 mmHg (*Figure 1A* and *B*). Given suspected BPVT, ECMO flow was reduced to increase native pulmonary flow. Repeat TOE 24 h later showed persistent severe obstruction with extensive thrombotic material within the left atrium, including mobile echodense components adherent to the prosthetic annulus and diffuse mural thrombotic involvement of the left atrium and surgically excluded left atrial appendage, measuring up to 10 mm in thickness, consistent with acute thrombotic prosthetic obstruction (*Figure 1C* and *D*).

Surgical reintervention and fibrinolysis were considered unsuitable because of recent surgery, ongoing VA-ECMO support, extensive left atrial and prosthetic thrombosis, and high bleeding and embolic risk. After multidisciplinary discussion and family consent, rescue transseptal PMBV was undertaken on Day 3 of VA-ECMO support.

**Figure 2:**
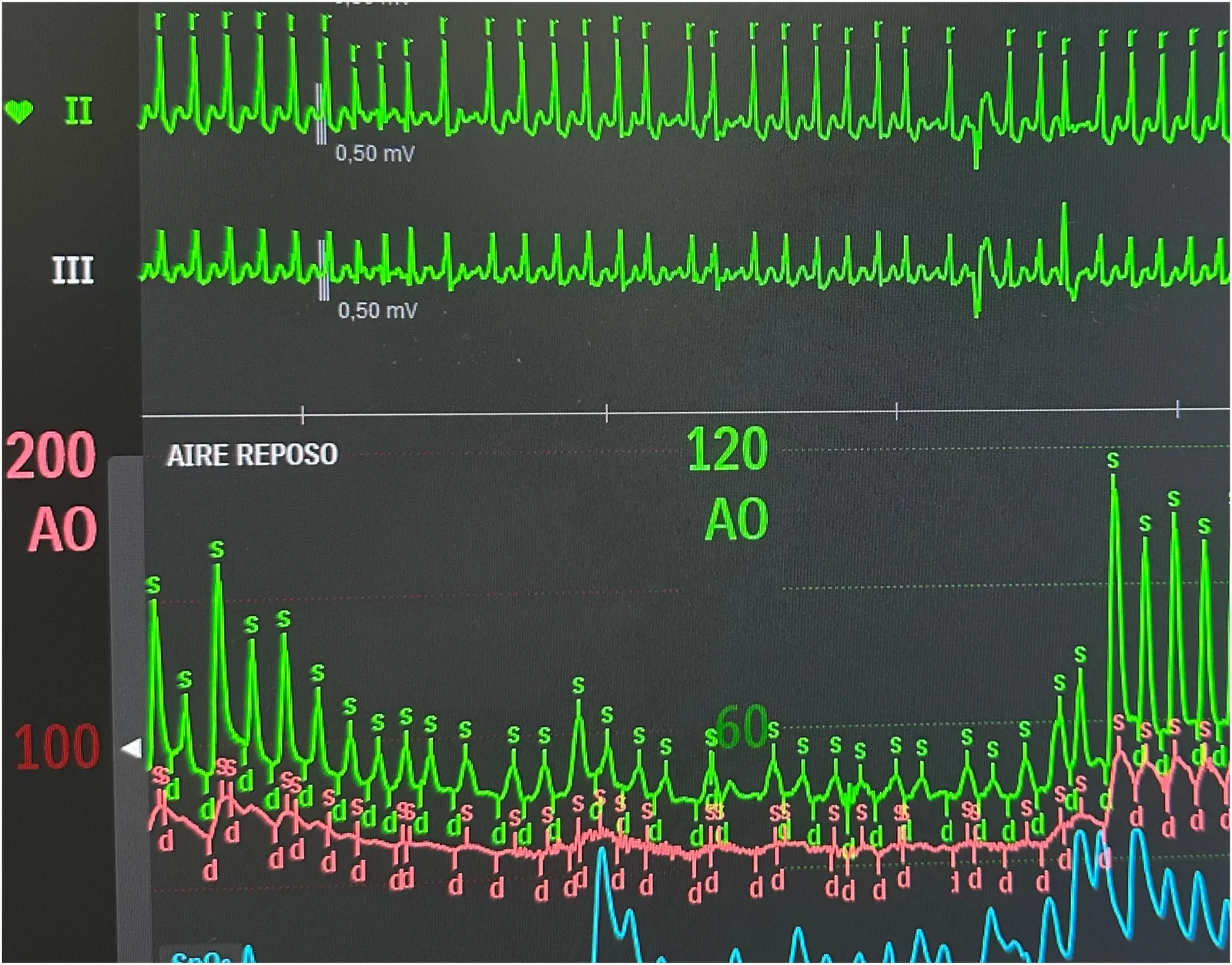
Haemodynamic tracing during balloon inflation. Simultaneous left atrial and aortic pressure recordings during balloon inflation show transient systolic equalization, consistent with effective relief of prosthetic mitral obstruction. The electrocardiographic tracing demonstrates atrial fibrillation.

Left femoral venous access was obtained for transseptal intervention. Cerebral embolic protection (Sentinel, Boston Scientific) was deployed via the right radial artery in an attempt to reduce cerebral embolic risk, despite technically challenging advancement related to post-surgical right axillary stenosis. Transseptal puncture was performed under TOE guidance, with the puncture site selected away from visible thrombus. Balloon dilatation of the bioprosthetic mitral valve was then performed using a 26-mm Inoue balloon with a single inflation (*Figure 1E*). Immediate TOE demonstrated improved bioprosthetic leaflet mobility, no significant mitral regurgitation, and a reduction in mean transmitral gradient from 25 to 4 mmHg (*Figure 1F*). Haemodynamic tracing showed transient systolic equalization of left atrial and aortic pressures during inflation (*Figure 2*). Given the very high embolic risk, immediate post-procedural cerebral angiography was performed to exclude acute cerebrovascular complications at that time and allow prompt endovascular treatment if needed. No angiographic evidence of immediate large-vessel cerebral embolization was identified.

Over the following 48 h, haemodynamic recovery allowed reduction of vasoactive support. No neurological deficits or peripheral embolic complications were observed. He was successfully weaned from VA-ECMO, transferred to the cardiac surgery ward, and discharged on post-operative Day 25 on acenocoumarol.

At 6-month follow-up, the patient remained stable. Transthoracic echocardiography demonstrated a normally functioning mitral bioprosthesis with a mean transmitral gradient of 4 mmHg and no pathological regurgitation. Left ventricular ejection fraction was 45%, right ventricular systolic function remained mildly impaired, and only mild tricuspid regurgitation was present. However, as follow-up TOE was not performed, residual thrombotic material cannot be definitively excluded.

## Discussion

Early BPVT should be suspected in patients who develop abrupt prosthetic mitral obstruction during VA-ECMO support. In postcardiotomy shock, low native forward flow and left atrial stasis create a highly thrombogenic milieu despite systemic anticoagulation. Prosthetic valve thrombosis during extracorporeal life support has been increasingly reported, particularly in patients with severe low-flow states.^2,3^ In our patient, abrupt early post-operative obstruction, restricted leaflet motion on TOE, concomitant extensive left atrial thrombus, and lack of improvement despite anticoagulation optimization strongly supported acute thrombotic prosthetic obstruction. Acute BPVT after mitral valve replacement, although rare, has been described.^1^

Standard treatment options may be unavailable in this setting. In this case, reoperation was prohibitive because of severe postcardiotomy shock, while fibrinolysis was considered unsafe due to recent surgery and substantial embolic and bleeding risk.

Manipulation of a thrombosed bioprosthesis in the presence of extensive left atrial thrombus carries a substantial risk of systemic embolization. However, TOE showed diffuse thrombotic involvement of the left atrium, prosthetic annulus, and surgically excluded left atrial appendage, rather than a large free-floating thrombus, making aspiration-based or staged thrombus-debulking strategies unlikely to adequately address the obstructive prosthetic component. The presence of extensive left atrial thrombosis further increased the procedural risk; however, this was considered acceptable given the otherwise near-fatal prognosis associated with persistent prosthetic obstruction during VA-ECMO support. As neither surgical reintervention nor fibrinolysis represented a viable option, rescue PMBV was performed following multidisciplinary discussion. Its efficacy likely resulted from disruption of fresh thrombotic material restricting leaflet motion, restoration of leaflet opening, and reduction of the transprosthetic gradient, thereby improving forward flow.^5,6^ In our patient, the immediate restoration of leaflet mobility and marked reduction in transprosthetic gradient, without significant regurgitation, support this mechanism.

There is no established evidence base or guideline-supported percutaneous strategy exists for acute BPVT. Published experience remains limited and largely confined to small case series of obstructed mechanical mitral prostheses treated with transcatheter leaflet release under cerebral embolic protection.^5,6^ A related scenario has been described in ECMO patients with dysfunctional mitral bioprostheses treated with balloon valvuloplasty.^7^ Therefore, in this case, PMBV should be considered an exceptional salvage manoeuvre rather than a standard therapeutic option.

Cerebral embolic protection was used in an attempt to reduce cerebral embolic risk, but it does not provide complete cerebral coverage and offers no protection against systemic embolization.^8^ Immediate post-procedural cerebral angiography was performed to exclude acute large-vessel cerebral embolization and allow immediate treatment if required; however, silent cerebral embolization cannot be excluded.

This single case cannot support routine use of PMBV for BPVT but expands the limited experience with transcatheter rescue approaches in catastrophic early BPVT when conventional treatment options are not feasible. Although the patient remained stable at 6-month follow-up, longer-term follow-up is warranted to assess durability and recurrent thrombosis.

## Patient’s perspective

A patient’s perspective could not be obtained because the patient was critically ill during the index hospitalization.

## Conclusion

Early recognition of prosthetic obstruction and timely multidisciplinary evaluation are crucial in patients with suspected early BPVT during VA-ECMO support. In exceptional circumstances, rescue transseptal PMBV may provide rapid haemodynamic relief and facilitate ECMO weaning when surgery and fibrinolysis are contraindicated or carry prohibitive risk.

## Lead author biography


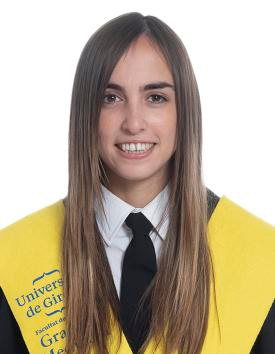
Aida Feu Masdemont is a cardiology resident at Hospital Universitari Dr. Josep Trueta de Girona, Spain, a centre with extensive experience in the care of critically ill cardiac patients. During her training, she has developed a particular interest in cardiac intensive care, haemodynamic instability, and the multidisciplinary management of complex cardiovascular disease. Alongside her clinical work, she is committed to ongoing education, clinical research, and scientific writing, with a focus on improving care for high-risk cardiac patients.

## Data Availability

The data underlying this article are available in the article.

## Artificial intelligence

The authors disclose the use of ChatGPT (OpenAI) for language editing and manuscript preparation. All content was reviewed and validated by the authors, who assume full responsibility for the final manuscript.
